# The Prophylactic Value of Using Nitrous Oxid-Oxygen in the Removal of Diseased Teeth to Avoid Systemic Reactions*Read before the Special Session on Anesthesia for Oral Surgery and Dentistry, of the Joint Meeting of the Mid-Western Association of Anesthetists and the National Anesthesia Research Society, at Kansas City, October 24-25, 1921. Reprinted from *Current Research in Anesthesia*.

**Published:** 1922-01

**Authors:** B. H. Harms

**Affiliations:** Omaha, Neb.


### THE PROPHYLACTIC VALUE OF USING NITROUS OXID-OXYGEN IN THE REMOVAL OF DISEASED TEETH TO AVOID SYSTEMIC REACTIONS*

B. H. HARMS, D.D.S., OMAHA, NEB.

Although the prevention of auto-inoculation during the removal of diseased teeth is something to be greatly desired, many years may pass before this goal is achieved in routine practice. In the meanwhile, the practitioners' time will have to be devoted to the prophylaxis of these conditions by such means as he has already at hand.

Even now, as in the past, anesthetics, local or general, are being considered only as means to an end, without due appreciation of their physiological and pathological action, immediate or remote, upon the various structures of the body.

* Read before the Special Session on Anesthesia for Oral Surgery and Dentistry, of the Joint Meeting of the Mid-Western Association of Anesthetists and the National Anesthesia Research Society, at Kansas City, October 24-25, 1921. Reprinted from *Current Research in Anesthesia.*

### COMPARATIVE CLINICAL RESULTS OF NITROUS OXID-OXYGEN AND PROCAIN ANESTHESIA

In view of this fact, I am offering for your consideration my comparative clinical experience in the removal of diseased teeth under general nitrous oxid-oxygen anesthesia and under procain local and block analgesia.

Briefly, the result of my clinical experience has been, that when I use a local anesthetic for the removal of one or more infected teeth the patient gets a more or less violent reaction or exacerbation of the existing infectious systemic symptoms, whereas, when using nitrous oxid-oxygen as the anesthetic, such reactions or exacerbations have been absent or slight, even though a larger number of teeth have been removed, and the technic of the operation in either instance has been the same. On the one hand, auto-inoculation from the extraction of one diseased tooth under local analgesia has been violent enough to place a seemingly strong, robust, patient in bed in the hospital, while, on the contrary, I have taken a bedridden patient out of bed in a hospital and under nitrous oxid-oxygen anesthesia have removed all of the teeth (sometimes twenty, or more) from the mouth, with a great amount of root-end and peridental pathology presenting, without any noticeable reaction or exacerbation. Having carried out this procedure in many patients, and checked up results with hospital records of these cases, it certainly seems that the choice of anesthetics in the extraction of diseased teeth is entitled to more consideration than it has been given in the past.

### CAUSATIVE FACTORS UNDERLYING AUTO-INOCULATION

In order to clarify my contention that local anesthetics are causative factors in precipitating the mentioned auto-inoculations, with their violent reactions or exacerbations, it will be necessary for me to refer rather extensively to the research work of McConnell, Rosenow, and others, who have delved so deeply into the pathology, bacteriology and therapy of focal infections, besides quoting also from certain text-books on pathology.

My clinical experiences, along these lines, have led me to believe that the interference of the local anesthetics on the circulation of the blood has more to do with the violent reaction or exacerbation, which follows the operation on infected areas, than any other factor. I do not believe that the small amount of local anesthetics used in these cases has enough toxicity to account for the striking results so routinely noted.

It is a well-known fact [4] that there are numerous bodily activities which, altogether, furnish a greater or lesser degree of what is called immunity. All types of bacteria, after gaining entrance into the body, do not cause disturbances in the same way. Certain organisms produce typical soluble toxins, which, by their absorption, cause serious disease. Others will form no toxins while living, but after death will break down and set free various poisonous products, which, in turn, affect the invaded individual. Some bacteria, simply by sheer numbers, may so overwhelm the body defenses as to cause death.

As every infected person does not die, there must be some form of natural protection.

*Phagocytosis.*—Many years ago, Metchnikoff came to the conclusion that the most important factor in the resistance to disease lay in the activity of certain cells of the blood, the polymorphonuclear leucocytes. Experience since then has shown that a very definite course of events takes place when infection by various organisms, particularly staphylococci and streptococci occurs. At first the changes are chiefly in the blood vessels, but shortly it will be noticed that in the tissues where the bacteria lie many leucocytes will be present. Further examination will show that, in many instances, bacteria will be found in the body of the cells. On account of this property, such leucocytes are known as phagocytes, this term referring to their ability to take up foreign substances. This activity depends upon the power they possess of independent activity similar to that of the ameba.

For a long time it was believed that this ability to engulf invading organisms depended entirely upon the activities of the leucocytes. That, as a result of some vital power, known as positive chemotaxis, the phagocytes were attacked by the invader. This force, it would seem, could act even through the walls of the smaller blood vessels and cause the leucocytes to leave their normal surroundings. It was noted, however, that, although positive chemotaxis was the usual phenomenon, there were times when the phagocytes seemed indifferent to certain bacteria. At one time it was thought that the leucocytes could take up dead bacteria only, and that it was supposed to be the reason why organisms were not always found in the cell. It has been demonstrated many times that these phagocytes can take up living organisms and this explains some cases of secondary infections distant from the primary focus.

Certain animals [5] are equally resistant to invasion by bacteria which can produce the most deadly disease in others; apparently because their defensive phagocytes are so active that long before the bacteria can gain proper foot-hold they are seized upon and devoured.

The immunity is not absolute, however, for influences such as exposure to cold or heat, great fatigue, anesthetics, or other illnesses, may so break down the resistance that, after all, even the resistant animal succumbs to infection.

In all the ills produced by living invaders there exists a struggle for supremacy, even for existence, between the host and the parasite, in which the stronger prevails and in which defenses are developed not only by the host but by the invader as well.

It is doubtless in this way that the bacteria accustom themselves, in passing from the body of one animal to that of another, to the action of the defensive reactions of the host, and thereby become more virulent.

For a time there were those who maintained that activity of the phagocytes constituted practically the whole defensive armament; while others, enthusiastic over their new discoveries, were just as sure that the neutralizing substances in the body fluids were all important, but now a reconciliation of these cellular and humoral doctrines has been effected, because it has been shown that they are very largely interdependent; phagocytosis depending upon the presence of auxiliary substances in the plasma, while, in turn, the leucocytes are important in producing other defensive fluid substances.

*Serum Defenses.*—By means of phagocytosis in normal animals, as has been explained, bacteria and other parasites are taken up and digested by the phagocytes; but the help of the serum of the animal is most important, for without it the leucocytes engulf very few bacteria. Denye, Wright, and others, have shown that the serum contains opsonins which act on the bacteria and prepare them for attack by the leucocytes. Neufield found that in the serum of animals, immunized by repeated introduction of certain bacteria, somewhat more resistant bodies (bacteroteopins) appear, which exercise the same function in making bacteria more approachable for the leucocytes. Aided by these substances, which bind themselves to the bacteria, or foreign cells, but do not affect the phagocytes, the wandering cells exhibit a greater phagocytic activity. Although the swallowing up of a bacterium does not necessarily kill it, it commonly does so, and at any rate removes it for the time being from the field of activity.

Doubtless a few bacteria brought thus into the most intimate relations with the body surfaces pass into the real interior, that is, into the tissues themselves, and are rapidly overcome and destroyed by phagocytic cells and by the destructive action of the blood and tissue fluids. Were it not for these defenses every one would quickly die from infection, since infection does occur with fatal results.

SELECTIVE ANESTHESIA IN PREVENTING AUTO-INOCULATION

Members of the dental and medical professions have, for some years, been taught that when a large number of diseased or infected teeth were to be removed that it was, or is, dangerous to remove too many at one sitting; the theory being that too large a number of bacteria, or too great quantities of toxin, are set free in the invaded individual and that they may so rapidly overwhelm the body defenses as to cause death.

In my clinical experience such untoward results have not occurred when the choice of the anesthetic has been given due consideration. An imperilling or disastrous result may, to a large extent, if not entirely, be prevented by the proper selection and use of the anesthetic.

Irons says that the resistance[3] of tissues themselves to the invading bacteria will also be a factor in determining whether the bacteria are quickly killed, or begin to grow. Thus bruised tissue, and tissue previously injured by disease may be less resistant than normal tissues, and may, owing to interferences with blood supply, offer conditions of oxygen tension different from normal tissue and, thereby, be a more favorable culture medium. It seems quite reasonable to suppose that changed environment of bacteria, either in the body or in the culture tube, may lead to changes in their growth requirements so that the same conditions under which they are barely able to survive and to produce low grade, almost symptomless lesions in the body, may later allow of greater growth and more serious lesion in the host, or, on the other hand, preclude any growth whatever.

In using local anesthetics for an operation on infected tissues where the anesthetic, to a greater or lesser extent, interferes with the circulation to the part to be operated on, either by infiltration or nerve-block, the same conditions are produced artificially as exist in bruised tissue, namely, interference with the circulation and oxygen tension, and, thereby, lowered resistance of that tissue to invading bacteria.

The only injections for blocking the branches of the fifth nerve, which do not interfere with the circulation of the blood in the parts of the mouth which are anesthetized, are the injections into the Gasserian ganglion and the spheno-palatine ganglion; all other injections, and especially those which are used in conjunction with infiltration, do interfere with the circulation to the parts which they anesthetize. True, a lower incisor could be anesthetized by blocking the inferior dental nerve on each side, which injection would in no way interfere with the circulation of the blood in the region of the lower incisor and would anesthetize the entire lower jaw with the exception of the buccal soft tissue region of the lower second and third molars, which is supplied by the long buccal nerve, and would also anesthetize the tongue, but to carry out a procedure such as this would be as uncalled for as a spinal anesthesia for a circumcision.

A bloodless field for the removal of teeth has been the aim of many operators and the condition very much desired by men who are using local anesthetics. While a bloodless field is a great help to an operator, it, on the other hand, is of great detriment to the patient, first, because it lowers the resistance of that tissue to invading pathogenic micro-organism, and, second, because it interferes with the normal coagulation time of the blood, which is nature's protection for any wound.

In a personal communication, Prof. Martin H. Fischer,[6] of the University of Cincinnati Medical College, who has so brilliantly investigated the relation of deoxygenation and edema to all conditions of systemic infection, writes that:

"I know that the contentions of Dr. Harms are absolutely right, and I know this from personal experience with many ill patients and dental operators of unusual skill. Where local anesthetics are used I make the operator cut down the amount of adrenalin used to the minimum and also the total amount of local anesthetic, trying to get him to inject the anesthetic where it will hit a nerve trunk. In other words, *produce the maximum of anesthesia with a minimum of local ischemia.* Of course, nitrous oxid-oxygen anesthesia is the anesthetic of choice. The trouble is that the dental operators, either do not know how to use it properly and do not get enough time, or else are bad operators from the start and so do not make a good job of an infected area in the briefer time of unconsciousness and insensibility to pain allowed them with nitrous oxid-oxygen. I, too, have seen bad systemic infections follow what I call these bungled mouth operations. They are very common, too, when operations are done badly on the tonsils, and here it is the same story. Unless local anesthetics are most carefully used and trauma is reduced to a minimum, there is much subsequent swelling of the injured part and great liability to infection. * * *

"I do not know exactly, of course, why the infection occurs more easily in the injured tissues, but I am inclined, personally, to the view that the lack of oxygen in the swollen tissues, aided, of course, by the good culture ground of blood and bruised tissues, tells the whole story. *Nearly all the organisms that produce these changes are partial tension organisms, and to put them into a state of lowered oxygen tension is to give them the advantage of development over that of the host.* It is this principle that I have made use of so often in treating the general joint-muscle and other infections with streptococcus. I think that the immune body development business has very little to do with getting these patients well. All you have to do is to shrink their swollen tissues, let oxygen get at the bacteria and they die. What is the best, for instance, for the treatment of *rheumatism* is air, caffeine and alkali, and wet dressing with magnesium sulphate, and what does all this mean, excepting systemic alkalinization, improvement in oxygen supply to the affected parts, and natural death of the infecting organisms?''

Teeth which have root-end pathology, such as a granuloma, are more or less walled off by nature from the surrounding tissue, and it is desirable that when these teeth are removed, as little auto-inoculation as possible should be given to the surrounding healthy tissue and the healthy tissue should not be placed in a position of lowered resistance.

Regardless of what surgical technic is employed for the removal of these teeth and their granuloma, is it reasonable to suppose that if the surrounding tissue is left in its highest state of immunity that the so-called reaction or exacerbation will be lessened?

In those fatal cases which have been reported because of having too many teeth removed at one sitting in only one case has any mention been made of the anesthetic used, and in that case the anesthetic was ether, and it was admitted that the patient had nephritis to begin with, which was, no doubt, aggravated by the ether anesthesia and not the removal of the infected teeth.

### CONCLUSIONS

(1) For the well-being of the patient anesthetics are not properly considered as to their physiological and pathological action.

(2) In the removal of diseased teeth it is desirable that none of the activity of the body defenses be limited or destroyed by the use of any drugs or anesthetics.

(3) The leucocytes are the body's first line of defense against invading micro-organisms.

(4) Any agent which limits the action of the leucocytes, either by destroying them or preventing their passage to the invaded part by initial ischemia and secondary edema, tends to lower the resistance of that tissue and the individual to the invading micro-organism.

(5) Any tissue which has had its normal circulation and oxygen tension interfered with has lost its normal degree of immunity.

(6) Local anesthetics initially interfere with the circulation, and oxygen tension of the parts that they anesthetize, and, secondarily, cause edema, thereby increasing the susceptibility of the part to infection and auto-inoculation.

(7) General anesthetics, other than nitrous oxid-oxygen, lower the patient's resistance to infection.

(8) Fatal cases from the removal of infected teeth have not been properly reported.

1222 First National Bank Bldg.

REFERENCES

[1] Edward C. Rosenow: The Relation of Dental Infection to Systemic Disease. *Dental Cosmos*, May, 1917.

[2] Edward C. Rosenow: Studies on Elective Localization: Focal Infection with Special Reference to Oral Sepsis. *The Journal of the National Dental Association*, November, 1919.

[3] E. E. Irons, M.D.: Discussion of Edward C. Rosenow's Paper. *The Journal of the National Dental Association*, November, 1919.

[4] Guthrie McConnell: The Principles of Bacterin (Vaccine) Treatment. *Dental Facts*, August, 1920.

[5] MacCallum: Text-Book of Pathology.

[6] Personal Communication from Prof. Martin H. Fischer.

